# Overweight and Its Associated Factors among Women of Reproductive Age in Dire Dawa, Eastern Ethiopia, 2021: Community-Based Cross-Sectional Study

**DOI:** 10.1155/2022/7268573

**Published:** 2022-08-16

**Authors:** Ismael Omer, Tariku Derese, Yitagesu Sintayehu

**Affiliations:** ^1^Department of Nutrition, Sitti Medical and Business College, Dire Dawa, Ethiopia; ^2^Department of Public Health, College of Medicine and Health Sciences, Dire Dawa University, Dire Dawa, Ethiopia; ^3^Department of Midwifery, College of Medicine and Health Science, Dire Dawa University, Dire Dawa, Ethiopia

## Abstract

**Background:**

Overweight in women of reproductive age is a major public health concern in developing countries because of overconsumption of low-quality food. Currently, being overweight is a major health concern worldwide. It exposes humans to various health problems. In Ethiopia, despite the trend indicated increasing in overweight, priority is given for undernutrition. In Dire Dawa, there is scarce evidence regarding reproductive age overweight. Therefore, this study is designed to assess the prevalence of overweight and its associated factors among women of reproductive age in eastern Ethiopia.

**Methods:**

A community-based cross-sectional study was conducted from May 15 to June 15, 2021, in Dire Dawa, Eastern Ethiopia; a multi-stage systematic sampling technique was used to select 559 women aged 15–49 years. Data were collected through face-to-face interviews using a structured pretested questionnaire. Ninety-five percent CI was used to identify the factors associated with overweight while controlling for all possible confounders using multivariable logistic regression. Statistical significance was set at a *P*-value of 0.05.

**Results:**

The results of this study revealed that the prevalence of overweight was 63.1% (95% CI: 59.0, 67.2). Overweight was significantly associated with weekly discretionary calories (AOR = 3.964, 95% CI (1.131, 13.894)), contraceptive use (AOR = 2.838, 95% CI (1.443, 5.580)), and monthly family income (AOR = 3.916, 95% CI (1.352, 11.340)).

**Conclusion:**

Overweight among women of reproductive age was high in Dire Dawa city. Discretionary calories per week, family monthly income, and contraceptive use were significantly associated with overweight. Developing and implementing community-based culturally sensitive, feasible, and potentially high-impact intervention to address the modifiable risk factors among women of reproductive age is critical.

## 1. Introduction

Overweight is a major health problem worldwide. The global prevalence of overweight has been significantly increasing over the past four decades [1]. Overweight and obesity cost the world billions of dollars a year in lost opportunities for economic growth and lost investments in human capital associated with the increased preventable morbidity and mortality rates in both children and adults [2]. In 2016, over 1.9 billion people aged 18 and older were overweight, and in 2016, 39% of adults aged 18 and over were overweight. Overweight is increasing at an alarming rate worldwide and has become a global epidemic [3].

Noncommunicable diseases related to overweight are global problems and challenges that are expanding worldwide in the twenty-first century, in terms of both human suffering and the damage they impose on the socioeconomic fabric of vulnerable countries, particularly low- and middle-income countries. They were responsible for an estimated 38 million (68%) of the world's 56 million deaths in 2012 [4, 5].

Chronic diseases will account for almost three-quarters of all deaths worldwide by 2020, with 71% of deaths from CVD and 70% of deaths from diabetes projected to occur in developing countries [6]. Being overweight or obese is associated with a high comorbidity. It is of great public health concern, particularly for cardiovascular diseases, type 2 diabetes, high blood pressure, high blood cholesterol, high triglycerides, certain types of cancer, and sleep apnea [7, 8].

Almost all countries face endemic overweight and obesity. However, the variations between and within countries differ significantly. Previously, it was thought that being overweight and obese were common problems in developed countries: however, recent studies conducted in developing countries have shown that being overweight is more common than being underweight in most countries [9]. Currently, an average of 20–50% of the urban population in Africa is either overweight or obese. In West Africa, the rate of overweight or obesity is approximately 10%. It was 3 times higher in women than that in men. In parts of West Africa, these rates have more than doubled over the past 15 years. In Morocco, more than 40% of the population is overweight, and three-quarters of the obese population worldwide will be in nonindustrialized countries by 2025 [10, 11].

According to the 2016 Ethiopian Demographic survey, the national prevalence rate of overweight or obesity is 1.5% among women, although the prevalence of overweight or obesity varies by region. The proportion of women, who are overweight or obese, which is indicative of overnutrition, has increased during the same period. The proportion of women who are overweight or obese has increased from 3% in 2000 to 8% in 2016 [12]. The prevalence of overweight among urban Ethiopian women was 12.1%. However, the prevalence of child overweight and obesity were 14.7%, which is higher than in adults and among psychiatry patients in Dire Dawa. The magnitude of overweight/obesity was significantly higher in the severely ill psychiatric groups (43.8%) than in the nonexposed controls (20.80%). The prevalence of overweight obesity was highest in major depressive disorders (40%), followed by schizophrenia (32%), and bipolar disorder (28%) [11, 13–15].

The fundamental cause of overweight is an energy imbalance between the calories consumed and expended. An increased intake of energy-dense foods, sedentary lifestyles, nature of work, and increasing urbanization have led to a global epidemic of overweight and obesity [16].

Factors contributing to overweight are foods that are high in fats, sugars, energy-dense foods, and increasing low-intensity activities due to new modes of transportation and rapid urbanization, which continue to cause dramatic changes in living environments as well as in diets and lifestyles that promote positive energy balance [17].

Although different studies indicate the existence and drastic progression of problems in the administration, there is a scarcity of research in our country, specifically in the Dire Dawa city, showing the prevalence and factors associated with overweight among women of reproductive age. Therefore, this study aimed to assess the prevalence of overweight and its associated factors among women of reproductive age in Dire Dawa, eastern Ethiopia.

## 2. Methods and Materials

### 2.1. Study Setting and Period

This study was conducted in the urban areas of Dire Dawa, eastern Ethiopia. The Dire Dawa Administrative Region is located in eastern Ethiopia, 515 km from Addis Ababa. According to the city administration health bureau report of 2007 [18], primary healthcare coverage is 100% in rural areas and 90% in urban areas. There were 6 hospitals (2 public, 3 private, and 1 Ethio-Djibout). Dire Dawa has 15 health centers and 34 health posts. It has 9 town kebels and 38 rural kebeles (kebele is a small administrative unit in Ethiopia), and the population for the 2013 budget year was estimated to be 521,000. Of these, 49% were men, and 51% were women. Women in reproductive age were estimated around 127,025 (24.4%)) in 2013 EFY. Dire Dawa is a desert, and most food groups are imported to urban areas from different parts of the country, mostly from the Oromia and Somalia regional states throughout the year. This study was conducted from May 15 to June 15, 2021.

### 2.2. Study Design

A community-based cross-sectional study design was employed among women of reproductive age (15–49 years) [19] in the Dire Dawa city. Based on national reproductive age classification, reproductive age group is from 15–49 years). However, all participants in this study were above 20 years.

### 2.3. Source and Study Population

All women of reproductive age (15–49 Years) [19] in Dire Dawa city are the study population. All women of reproductive age randomly selected from the selected households in each kebele (small administrative units in Ethiopia) in Dire Dawa city are the target population.

### 2.4. Eligibility Criterion

All women of reproductive age (15–49) who were permanent dwellers in Dire Dawa city and were willing to participate were included in this study. Pregnant women (women's weight increases physiologically during pregnancy) and those who are unable to communicate effectively during the data collection period were excluded from this study. The pregnancy status of women was identified by asking the women before data collection started.

### 2.5. Outcome and Predictors Variable

Overweight is the outcome variable of this study. The predictors variables include all sociodemographic variables; age, religion, ethnicity, educational status, marital status, parity, dietary habit, physical activity, and socioeconomic status.

### 2.6. Operational Definitions

  Body mass index (BMI): it is a measure of weight adjusted for height, calculated as weight in kilograms divided by the square of height in meters (“kg/m^2^”) [20].
  Dietary behavior: in this study, we referred to the participants' usual foods [20].
  Moderate physical activity: in this study, any activity that required moderate physical effort and slight increases in breathing or heart rate was considered moderate physical activity [20].
  Overweight: overweight in this study was defined as a BMI of >23.1 kg/m^2^.
  Physical activity: in this study, physical activity is defined as any bodily movement engaged during work, transport, and leisure time that results in increased energy expenditure. It also includes exercise and sports, household chores, and other activities of daily living [20].
  Permanent residents: in this study, people had lived in the study area for the previous six months.
  Reproductive age: in this study, women were defined as those aged between 15–49 years [20].
  Sedentary behavior: in this study, the time spent by women sitting, reclining, or performing activities that require minimal or no physical movement or engagement was recorded [20].
  Vigorous physical activity: this is defined as any activity that requires hard physical effort and leads to a high increment in breathing and heart rate [20].

### 2.7. Data Sources or Measurements

This study used two types of measurement tools: questionnaires and anthropometric measurements. A structured questionnaire was used to collect the data; most of the questionnaire items to assess global physical activities and food dietary intake were adapted from the WHO STEP tool for chronic disease risk surveillance [21–23], and some modifications were made in accordance with the local situation and study objective. Data were collected by eight health extension workers and supervised by health professionals during data collection. Data collectors and supervisors were trained in the objectives, methods, and materials used in the research. Interviewing techniques, anthropometric measurements, and data recoding before the actual task of data collection were demonstrated during training. The questionnaire was divided into four parts (socioeconomic and behavioral, dietary intake, global physical activity, and anthropometry), which were used to determine the nutritional status of women of reproductive age (15–49 years).

### 2.8. Measurement of Overweight

There are a wide variety of methods for measuring body weight. The most commonly used method for adult anthropometric measurements is the BMI method. BMI is a simple index of weight-for-height that is commonly used to classify underweight, overweight, and obese adults. It is defined as the weight in kilograms divided by the square of the height in meters (kg/m^2^). A BMI cut-off point for diagnosing overweight and undernutrition (chronic energy deficiency) is obesity: > 24.5, overweight: 23.1–24.5, and normal: 21.9–23 [24]. However, in this study, we considered overweight BMI greater than 23.1.

### 2.9. Data Collection Procedure

The data collectors went to households that had been identified during sampling and tried to contact the eligible people (all women of reproductive age “15–49 years”). The reasons for the visits were explained to the participants. Eligible participants were requested to participate in this study. A pretested structured questionnaire and anthropometric measurements were used to collect data. The sociodemographic part asked the respondent about their age, marital status, parity, and socioeconomic status. The physical activity part was interviewed; physical activity data, type, frequency, duration, and intensity of physical activity during work, transportation, and leisure time in a typical week. In addition to this, dietary questions such as frequency of meals, favorite foods, vegetable and fruit consumption, and so on. were also asked. Lastly, the data collectors performed height and weight measurements. Height was measured using standard procedures with a portable height scale to the nearest 0.5 cm. Weight was recorded to the nearest 0.1 kg using a digital scale. The subjects were weighed while wearing light clothing.

### 2.10. Assurance of Quality

The questionnaires were pretested a week before the actual data collection day for 5% of the sample size in the same sample unit (households) of the study but in a kebele that was not selected for the study. The original English version of the questionnaire was translated into Amharic and Somali versions to avoid ambiguity in the concept of the question, and then the local version was translated back into English by a professional to check its consistency, and modifications were made accordingly. The principal investigator trained the data collectors and supervisor; during data collection, the trained supervisor checked in the field to see how the data collectors were carrying out their tasks and responsibilities. To avoid measurement bias in anthropometric measurements, the weight scale was calibrated to 0, on a daily basis before the start of the measurement. Both height and weight were measured twice for every subject; in cases where the two results were different; the average of the two was used. At the end of each data collection day, the principal investigator would also check the completeness of the filled questionnaires.

### 2.11. Sample Size Determination and Sampling Procedure

The sample size was determined using a single population proportion formula, and the proportion was obtained from a previous study in Ethiopia with *n* = (*Z*_/2_)^2^*p* [1-*p*]/*d*^2^, where *n* = required sample size; *Z*/2 = critical value for normal distribution at 95% confidence level, which equals 1.96 (*z* value at alpha = 0.05); *P* = prevalence of overweight (32.8%) [25]; and *d* = absolute precision (margin of error 5%). Using 1.5 design effect and 5% nonresponse rate, the total sample size of this study was 559.

In this study, a multistage sampling method was used, by considering Dire Dawa city, which consists of nine kebeles (small administrative units in Ethiopia). Four kebeles were selected from these nine kebeles (nine urban kebeles in Dire Dawa city). A total number of women of reproductive age from each kebele was taken from the family folders. A proportional study unit was selected by simple random sampling using the lottery method, followed by systematic sampling techniques. The calculated sample size (559) was proportionally allocated according to the women of reproductive age load for each kebele. A systematic sampling technique with a *k*^th^ interval for each kebele (kebele 01 (17), kebele 07 (8), kebele 08 (13), and kebele 09 (32)) was used to identify the study households from each kebele.

Based on total cases of women of reproductive age in each kebele, kebele 01 (152 women of reproductive age), kebele 07 (149 women of reproductive age), kebele 08 (130 women of reproductive age), and kebele 09 (128 women of reproductive age) were selected and included in this study. In cases where there was more than one eligible individual or woman of reproductive age (15–49) in the selected household, a lottery method was used to select one. In the event that there were no eligible persons in the selected household, the next door was visited.

### 2.12. Processing and Analysis of Data

Before data entry, the investigators checked data consistency and completeness. Using EpiData version 3.02, data were sorted, coded, and entered into a computer, and the data were cleaned by looking for errors, impossible or implausible values, and inconsistencies that could be due to coding or data entry errors. Then data were exported to the SPSS version 16 software package for analysis. Both descriptive (frequency and percentage) and analytical approaches were used to analyze the data. To examine the subgroups and variable interaction, variables found to be significant (*P* < 0.25) in the bivariate analysis were used to construct multivariate models. The fitness of the model was tested by the Hosmer–Lemeshow goodness-of-fit test. The multicollinearity effect was checked, and variables with SE > 2 were removed from the analysis. Odds ratios with 95% confidence intervals (CI) were estimated to identify factors associated with overweight while controlling for all possible confounders using multivariate logistic regression. The level of statistical significance was set at *P* < 0.05.

## 3. Results

### 3.1. Households' Sociodemographic Characteristics

A total of 542 participants were interviewed, with a 96.9% response rate. The majority of the respondents, 399 (73.6%), were in the age range of 20–24 years, with a mean and standard deviation (±SD) of 27.3 ± 5.6. Of the study participants, 242 (44.6%) were married. A total of 213 participants (39.3%) had a monthly family income of more than 10,000 ETB, and 42.6% had no children (Table 1).

### 3.2. The Prevalence of Overweight among Women of Reproductive Age in Dire Dawa, 2021 (*n* = 542)

The majority of the study participants were overweight (63.1%; 95% CI: 59.0 to 67.2) and 36.9% (95% CI: 32.8 to 41.0) had normal and mild-to-severe chronic energy deficiency.

### 3.3. Women's Dietary Consumption

The majority of participants in the study consumed 98.5% of vegetables and 92.4% of discretionary calories (baqlawa, mushabag, and halawa). Cereal consumers were 91% of the participants, while fruit consumers consumed it once a week. Meat consumers were 96.3%, and meat products accounted for 85.4% of the total respondents (Table 2).

### 3.4. Physical Activities of Women

The majority of the respondents (439 (81.0%)) did not engage in moderate-intensity activities. A total of 21.6% of respondents reported that they do moderately intensified sports (Table 3).

### 3.5. Factors Associated with Overweight among Women of Reproductive Age

The highest prevalence of overweight was observed among women who consumed discretionary calories (AOR = 3.964, 95% CI (1.131, 13.894)), compared to women who did not consume discretionary calories per week. Furthermore, family monthly income was significantly associated with overweight; the prevalence of overweight was higher among women who had a higher monthly income (AOR = 3.916, 95% CI (1.352, 11.340)) than those with a low monthly income. Using contraceptives was associated with overweight among women. Women who used contraceptives were two times more risks for overweight than those who did not use any contraceptive method (AOR = 2.838, 95% CI (1.443, 5.580); Table 4).

## 4. Discussion

The study was conducted to determine overweight among women of reproductive age and associated factors in Dire Dawa. The overall prevalence of being overweight was 63.1% (95% CI: 59.0, 67.2). This prevalence rate of overweight is higher than findings from a study conducted in Addis Ababa in which the prevalence of overweight/obesity increased significantly by 28%. Specifically, the prevalence of urban obesity increased by 43.3%, that is, from 3.0% to 4.3% in about 15 years [26]. In a systematic review and meta-analysis findings in Ethiopia, the prevalence of overweight was higher, 22.6% in studies published since 2015 and 22.4% in studies conducted only in urban settings [27], with the other finding in Ethiopia, The prevalence of overweight and obesity among the study participants was 11.5% and 3.4%, respectively. The combined prevalence of overweight and obesity was 14.9%. Of the total participants who are overweight or obese, 83.3% were urban dwellers, and the remaining 16.7% were rural dwellers [28]. The higher percentage of overweight among Dire Dawa city's women of reproductive age might be due to their sedentary life and higher adaptation of unhealthy dietary habits (energy-dense foods, sweets, fats, etc.) increasing locally made energy-dense foods such as Malawa, Baklaba, and Mushebak.

This finding is higher compared to different findings in Africa, Demographic and Health Survey (DHS) data collected in 32 sub-Saharan African countries indicated the pooled prevalence of overweight for the region was 15.9% (95% CI, 15.7–16.0) with the lowest in Madagascar 5.6% (95% CI, 5.1–6.1) and the highest in Swaziland 27.7% (95% CI, 26.4–29.0) [29], in Nigeria the prevalence of overweight were 18.1% [30] and in Zimbabwe the prevalence of overweight a 25% in 2005 to 2015 [31]. The variation may be due to food consumption pattern of the community, sedentary lifestyle, sociodemographic characteristics, and geographical and study time difference.

Regarding factors associated with overweight, using contraceptives was significantly associated with overweight among women who used contraceptives were 2.838 times more likely to have overweight than women who did not use any contraceptive method. This finding is similar to a finding in Kenya [32] and in Bangladesh [33]. The possible explanation may be hormonal side effects.

Furthermore, having a higher family monthly income was associated with overweight. Women having higher monthly incomes were 3.916 times more likely to have overweight as compared to women who have lower monthly incomes. This finding is similar to other studies in different parts of Ethiopia ([28]) [34]; Dar es Salaam, Tanzania ([35]); Zimbabwe [31]; and Kenya [32]. This might be due to their capacity to purchase more energy-dense foods, as witnessed by studies in both developed and developing countries that show high-income households purchasing foods from supermarkets. Traditionally, in Ethiopia, consuming high-fat foods was considered an indicator of better social status, and consumption of fatty food is a common phenomenon.

This study indicated that discretionary calories per week are associated with overweight among 20–49 years age of women. This finding is similar to other findings from Dar es Salaam, Tanzania [35].

## 5. Conclusion

The overall prevalence of overweight was very high among reproductive-age women in Dire Dawa city. Women who consumed discretionary calories per week, higher family monthly income, and contraceptive use were the factors associated with a high risk of overweight among women of reproductive age in Dire Dawa city. Developing and implementing community-based culturally sensitive, feasible, and potentially high-impact intervention to address the modifiable risk factors of overweight among women of reproductive age is critical.

### 5.1. Limitation of the Study

The cross-sectional nature of the survey does not allow the cause-and-effect relationship between independent variables and overweight. It is difficult to generalize to the entire country since data.

## Figures and Tables

**Table 1:** Sociodemographic characteristics among women of reproductive age in Dire Dawa, 2021 (*n* = 542).

Variables	Level	Frequency	%
Age (years)	20–24	203	37.5
	25–29	169	31.2
	30–34	101	18.6
	35–39	51	9.4
	40–44	15	2.8
	45–49	3	0.6

Marital status	Single	222	41.0
	Married	242	44.6
	Widowed	36	6.6
	Divorced	42	7.7

Educational status	Not able to read and write	166	30.6
	Able to read and write	109	20.1
	Primary education	125	23.1
	Secondary education	116	21.4
	College and above	26	4.8

Monthly family income (Birr)	2,000–5,000	122	22.5
	5,000–10,000	207	38.5
	Above 10,000	213	39.3

Parity	No child	231	42.6
	1–2 children	113	20.8
	3–4 children	102	18.8
	>4 children	96	17.7

**Table 2:** Dietary consumption among women of reproductive age in Dire Dawa, 2021 (*n* = 542).

Variables	Level	Frequency	%
Cereal consumed per week	>1 day per week	493	91.0
	No intake	49	9.0

Legumes consumed per week	1 day per week	373	68.8
	No intake	169	31.2

Vegetables consumed per week	1 day per week	534	98.5
	No intake	8	1.5

Fruit consumed per week	1 day per week	522	96.3
	No intake	20	3.7

Oils consumed per week	1 day per week	400	73.8
	No intake	142	26.2

Spices consumed per week	1 day per week	280	51.7
	No intake	262	48.3

Discretionary calories consumed per week	1 day per week	501	92.4
	No intake	41	7.6

Dairy products per week consumed	1 day per week	404	74.5
	No intake	138	25.5

Meat and meat products per week consumed	1 day per week	463	85.4
	No intake	79	14.6

Contraceptive use	Yes	214	39.5
	No	328	60.5

**Table 3:** Physical activities among women of reproductive age in Dire Dawa, 2021 (*n* = 542).

Variables	Level	Frequency	%
Moderate intensity activity	0 day	439	81.0
	>1 day	103	19.0

Biking	0 day	481	88.7
	>1 day	61	11.3

Moderate intensity sport	0 day	425	78.4
	>1 day	117	21.6

Moderate physical activity	0 hour	337	62.2
	1 hour	145	26.8
	2 hours	44	8.1
	>3 hours	16	3.0

Time spent sitting or rest per day	1 hour	61	11.3
	>2 hours	481	88.7

**Table 4:** Factors associated with overweight among the reproductive age group in Dire Dawa, 2021 (*n* = 542).

Variables	Overweight		COR (95% CI)	AOR (95% CI)	*P*-value
	Yes	No			
Family income (Birr)					
2,000–5,000	74 (60.6)	48 (39.4) 1			
5,000–10,000	115 (55.5)	92 (44.5)	0.811 (0.514, 1.278)	1.364 (0.597, 3.115)	0.461
Above 10,000	68 (82.9)	14 (17.1)	1.654 (1.034, 2.647)	3.916 (1.352, 11.340)	0.012^*∗*^
Contraceptive use					
Yes	182 (55.5)	146 (44.5)	1	1	
No	160 (74.8)	54 (25.2)	2.377 (1.629, 3.467)	2.838 (1.443, 5.580)	0.002^*∗*^
Discretionary calories per week					
1 day per week	322 (64.2)	179 (35.8)	1	1	
No intake	20 (48.7)	21 (51.3)	1.889 (0.997, 3.579)	3.964 (1.131, 13.894)	0.031^*∗*^
Fruit per week consumed					
1 day per week	331 (63.4)	191 (36.6)	1	1	
No intake	11 (55)	9 (45)	1.418 (0.577, 3.483)	0.297 (0.063, 1.399)	0.125
Meat and meat products per week consumed					
1 day per week	298 (64.3)	165 (35.7)	1	1	
No intake	44 (55.7)	35 (44.3)	1.437 (0.886, 2.329)	1.345 (0.624, 2.897)	0.449
Dairy products per week consumed					
1 day per week	70 (50.7)	68 (49.3)	1	1	
No intake	272 (67.4)	132 (32.6)	2.002 (1.351, 2.966)	1.993 (0.937, 4.239)	0.073
Oils per week consumed					
1 day per week	68 (47.8)	74 (52.3)	1	1	
No intake	274 (68.5)	126 (31.5)	2.366 (1.601, 3.499)	1.845 (0.896, 3.799)	0.096

^*∗*^Statistically significant at *P*-value *<*0.05 in multivariate logistic regression analysis.

## Data Availability

The data sets used and/or analyzed during the current study are available from the corresponding author on reasonable request.

## Abbreviations

BMI: Body mass index
CI: Confidence interval
CSA: Central statistical agency
CVD: Cardiovascular disease
EDHS: Ethiopian demographic health survey
NCD: Noncommunicable disease
SPSS: Statistical Package for Social Sciences
SSA: Sub-Saharan Africa
WHO: World Health Organization
AOR: Adjusted odd ratio
COR: Crude odd ratio.
